# Enhancing the Strength and Ductility Synergy of Lightweight Ti-Rich Medium-Entropy Alloys through Ni Microalloying

**DOI:** 10.3390/ma17122900

**Published:** 2024-06-13

**Authors:** Po-Sung Chen, Jun-Rong Liu, Pei-Hua Tsai, Yu-Chin Liao, Jason Shian-Ching Jang, Hsin-Jay Wu, Shou-Yi Chang, Chih-Yen Chen, I-Yu Tsao

**Affiliations:** 1 Institute of Materials Science and Engineering, National Central University, Taoyuan 320, Taiwan; 2 Department of Mechanical Engineering, National Central University, Taoyuan 320, Taiwan; 3 Department of Materials Science and Engineering, National Yang Ming Chiao Tung University, Hsinchu 300, Taiwan; 4 Department of Materials Science and Engineering, National Tsing Hua University, Hsinchu 300, Taiwan; 5 Department of Electrophysics, National Yang Ming Chiao Tung University, Hsinchu 300, Taiwan

**Keywords:** medium-entropy alloy, lightweight, non-equiatomic, solid solution strengthening, thermo-mechanical treatment

## Abstract

Medium-entropy alloys (MEAs) have attracted considerable attention in recent decades due to their exceptional material properties and design flexibility. In this study, lightweight and non-equiatomic MEAs with low density (~5 g/cm^3^), high strength (yield strength: 1200 MPa), and high ductility (plastic deformation: ≧10%) were explored. We fine-tuned a previously developed Ti-rich MEA by microalloying it with small amounts of Ni (reducing the atomic radius and increasing the elastic modulus) through solid solution strengthening to achieve a series of MEAs with enhanced mechanical properties. Among the prepared MEAs, Ti65Ni1 and Ti65Ni3 exhibited optimal properties in terms of the balance between strength and ductility. Furthermore, the Ti65Ni3 MEA was subjected to thermo-mechanical treatment (TMT) followed by cold rolling 70% (CR70) and cold rolling 85% (CR85). Subsequently, the processed samples were rapidly annealed at 743 °C, 770 °C, 817 °C, and 889 °C at a heating rate of 15 °C/s. X-ray diffraction analysis revealed that the MEA could retain its single-body-centered cubic solid solution structure after TMT. Additionally, the tensile testing results revealed that increasing the annealing temperature led to a decrease in yield strength and an increase in ductility. Notably, the Ti65Ni3 MEA sample that was subjected to CR70 and CR85 processing and annealed for 30 s exhibited high yield strength (>1250 MPa) and ductility (>13%). In particular, the Ti65Ni3 MEA subjected to CR85 exhibited a specific yield strength of 264 MPa·cm^3^/g, specific tensile strength of 300 MPa·cm^3^/g, and ductility of >13%.

## 1. Introduction

With the increasing awareness of operational costs and environmental concerns, manufacturers are increasingly prioritizing improvements in fuel economy to mitigate carbon emissions [1]. Therefore, the development and application of lightweight materials have become crucial for transportation and energy applications [2]. However, conventional lightweight materials, such as Al and Ti alloys, do not exhibit high strength. Conversely, high-strength steel exhibits exceptional mechanical properties; nevertheless, its high density substantially contributes to its structural weight, resulting in high fuel consumption during transportation [3]. Therefore, metallic materials with high specific strength (i.e., those with both high strength and low density) must be explored.

A novel alloy type called high-entropy alloys (HEAs), composed of multiple principal elements, was proposed to overcome the limitations of conventional alloying methods [4]. HEAs exhibit distinctive effects and can be synthesized with a wide range of alloy designs, resulting in superior properties compared with conventional alloys [5,6]. Owing to their excellent material properties, HEAs have been further modified to formulate non-equiatomic HEAs and medium-entropy alloys (MEAs) [7,8,9,10]. These alloys exhibit flexible designs while maintaining the distinctive characteristics of HEAs [11,12,13]. Some studies have proposed lightweight MEAs with excellent material properties, demonstrating the substantial potential of MEAs in industrial applications [14,15,16].

The applicability of alloys can be increased through solid solution strengthening, which enhances the mechanical properties of alloys. Microalloying, a method that involves the addition of small amounts of elements with smaller or larger atomic radii, effectively improves material strength through substitutional solid solution strengthening [17]. In addition, thermo-mechanical treatment (TMT) is an effective method for improving the mechanical properties of alloys [18,19]. Combining an annealing process with solid solution strengthening can also result in a finely tuned alloy microstructure, thereby enhancing the overall mechanical properties of the alloy [20,21,22].

In the previous study, we explored the quaternary Ti_65_(AlCrNb)_35_ MEAs with simple solid solution structures and excellent tensile mechanical properties [15]. Afterwards, the quinary Ti-rich MEAs with vanadium addition were proposed [23,24]. Moreover, the effect of thermo-mechanical treatment on Ti-rich MEAs were also investigated [25,26]. In this study, we fine-tuned the composition of a previously developed ductile Ti_65_(AlCrNbV)_35_ alloy [23,27] by microalloying it with small amounts of Ni (characterized by a smaller radius) through solid solution strengthening to produce a series of MEAs with enhanced mechanical properties. Additionally, TMT was employed to further modify the alloy’s microstructure in order to enhance its strength and ductility. Overall, this study developed a lightweight MEA (approximately 5 g/cm^3^) with high specific tensile strength (≧280 MPa·cm^3^/g) as well as high ductility (≧10%).

## 2. Experimental Procedure

### 2.1. Material Design

A series of Ti_x_(AlCrNbV)_100−x−y_Ni_y_ MEAs were synthesized using high-purity Ti (99.99%), Al (99.99%), Cr (99.99%), Nb (99.99%), V (99.99%), and Ni (99.9%). Master alloys were fabricated through arc melting in an Ar atmosphere and were subjected to remelting four times to ensure homogeneity. Subsequently, each of the alloy melts was cast into an ingot with dimensions of 40 mm × 20 mm × 10 mm through drop casting in an Ar atmosphere.

### 2.2. Thermo-Mechanical Treatment

Before TMT, the MEA samples were homogenized at 1000 °C for 2 h in a high-vacuum atmosphere (<10^−5^ Torr), followed by rapid water quenching. Subsequently, the samples were subjected to one of two different cold rolling processes, namely cold rolling 70% (CR70) and cold rolling 85% (CR85), at room temperature to accumulate the strain energy. The samples were then subjected to rapid annealing (at a heating rate of 15 °C/s) in a vacuum tube furnace under a pressure of 2 × 10^−5^ Torr for varying durations (30, 34, 41, and 63 s).

### 2.3. Microstructure Characterization

The density of the MEAs was calculated using Archimedes’ principle. The crystal structures of the MEAs were analyzed using an X-ray diffraction (XRD) instrument (D2, Bruker, Billerica, MA, USA) equipped with Cu K_α_ radiation. The scan speed was 0.06°/s and the step time was 1 s. The samples were sanded using silicon carbide sandpaper with grit sizes ranging from #80 to #2000. The microstructures of the MEAs were examined through optical microscopy (OM; BX51M, Olympus, Tokyo, Japan) and electron backscatter diffraction (EBSD; HKL Channel 5, Oxford Instruments, Hobro, Denmark). The samples were polished using an Al_2_O_3_ polish suspension with particle sizes of 0.3 and 0.05 µm before OM analysis. The samples were also polished using an electro-polishing machine before EBSD analysis. The surface fracture morphologies of the MEAs after being subjected to tensile loading were analyzed through scanning electron microscopy (SEM; F50 Inspect, FEI, Hillsboro, OR, USA).

### 2.4. Mechanical Testing

The hardness of the MEAs was assessed using a Vickers hardness tester (HV-115, Mitutoyo, Kawasaki, Japan) under a loading of 5 kg for 10 s. The MEAs were subjected to tensile tests performed using a universal testing machine (HT9102, Hung Ta, Taichung, Taiwan) under quasi-static loading with a strain rate of 1 × 10^−4^/s. The samples subjected to the tensile tests had dimensions of 5 mm (length) × 2 mm (width) × 1.5 mm (thickness).

## 3. Results and Discussion

On the basis of our previously developed quinary lightweight Ti_65_(AlCrNbV)_35_ MEA, we designed and prepared a series of lightweight Ti_x_(AlCrNbV)_100−x−y_Ni_y_ (TiXNiY) MEAs in the present study, as listed in Table 1. Notably, the configuration entropy of all the as-prepared alloys ranged from 9.67 to 10.64 kJ·Mol^−1^, complying with the definition of MEAs. Furthermore, the entropy of the MEAs increased slightly with Ni content. The atomic size difference (δr) calculated by Equation 1 of the MEAs also increased slightly with Ni content, with the δr values of all MEAs being approximately 5%. The value falls within 0% ≤ δr ≤ 6.6%, which is favorable for the formation of a single solid solution structure.

(1) $$\delta_{r}=\sqrt{\sum_{i=1}^{n}c_{i}{\left(1-r_{i}/\bar{r}\right)}^{2}}$$

### 3.1. Characterization of As-Cast Ti_x_(AlCrNbV)_100−x−y_Ni_y_ (TiXNiY) MEAs

The measured densities of the as-prepared alloys ranged from 5.02 to 5.12 g/cm^3^, similar to the theoretical densities calculated according to the mixing rule (Table 2). Furthermore, the densities increased slightly with Ni content. 

**Figure 1:**
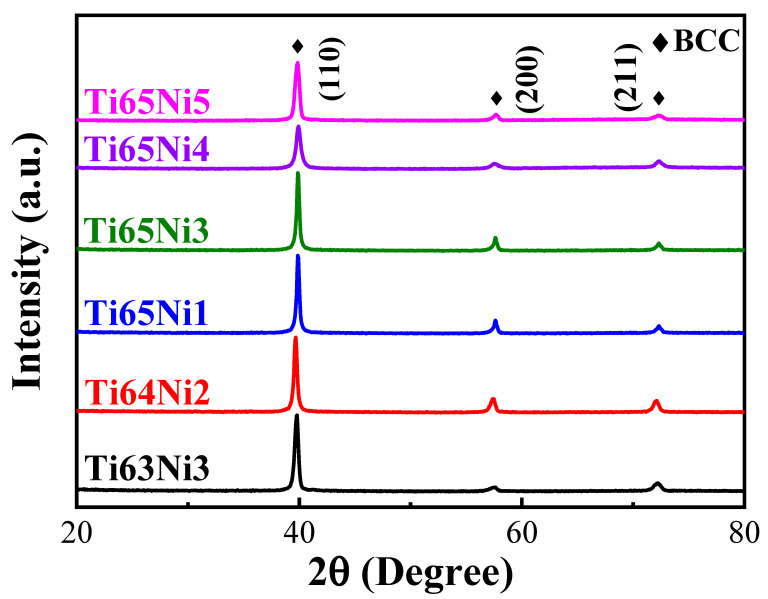
The XRD patterns of the as-cast Ti_x_(AlCrNbV)_100−x−y_Ni_y_ MEAs.

The XRD spectra of the MEAs revealed a single set of characteristic peaks, which could be attributed to the body-centered cubic (BCC) phase (Figure 1). Notably, a diffraction peak was observed, and it shifted toward the right as the Ni content increased; this could be attributed to the addition of Ni, which has a smaller atomic size (124 pm) [28]. By contrast, increasing the Ti content led to a leftward shift of the diffraction peak, which could be attributed to the larger atomic size (140 pm) of Ti. Similarly, the lattice constants of the MEAs calculated from XRD data decreased as the Ni content increased (Table 1).

**Figure 2:**
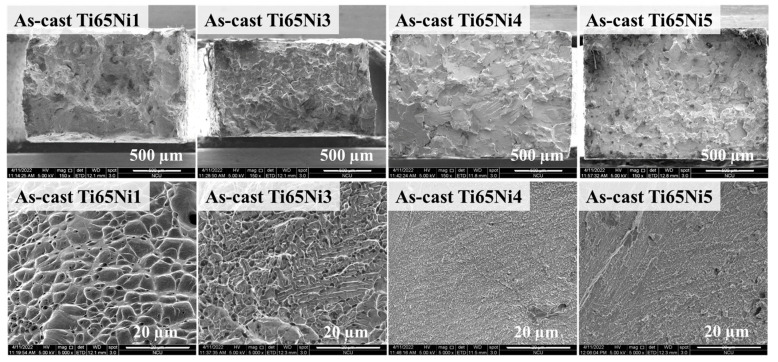
The SEM images of the fracture morphology of the as-cast Ti65Ni1, Ti65Ni3, Ti65Ni4 and Ti65Ni5 MEAs.

The hardness and tensile test results obtained for the MEAs are presented in Table 3. The results indicated that adding Ni effectively enhanced both the hardness and yield strength of the samples. Conversely, increasing the Ti content reduced hardness and yield strength of the samples. Since Ti possesses a lower Young’s modulus, a high concentration of Ti will soften the mechanical properties of the alloy, which is consistent with the previous study [15]. In addition, because the atomic radius of the Ni (124 pm) is less than the principal element of Ti (140 pm), high Ni content will severely distort the lattice, which restricts the propagation of dislocations, and consequently improves the strength of the MEAs. These findings thus indicate that Ni can effectively improve the mechanical properties of MEAs prepared through solid solution strengthening. Overall, among the as-cast MEAs, Ti65Ni3 exhibited the optimal combination of strength and ductility. Moreover, SEM image revealed that the Ti65Ni3 alloy specimen possessed a typical ductile fracture surface after being subjected to a large plastic strain (Figure 2). Conversely, Ti65Ni4 exhibited a clear, brittle fracture surface with cleavage facets before yielding, consistent with the mechanical properties of the MEA.

**Figure 3:**
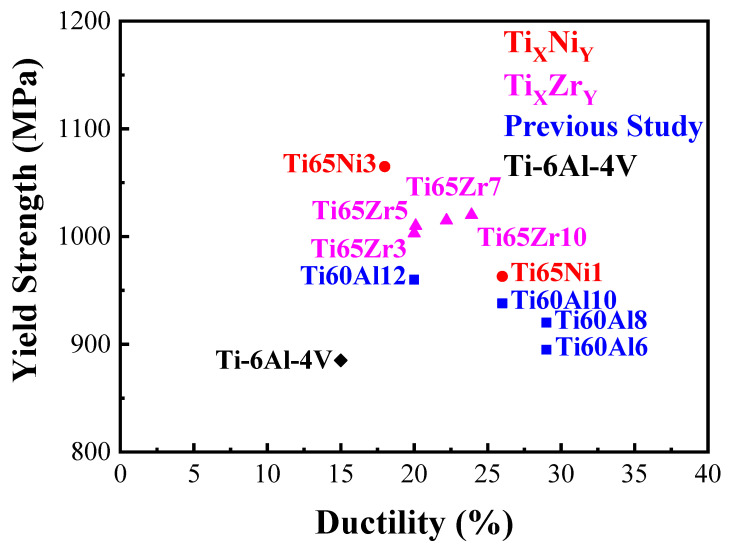
Comparison of yield strength and ductility for the as-cast Ti65Ni1 and Ti65Ni3 MEAs to the previous studies Ti_60_Al_x_(VCrNb)_40−x_ (x = 6, 8, 10, 12), Ti_65_(AlCrNbV)_35−x_Zr_x_ (x = 3, 5, 7, 10) and as-cast commercial Ti-6Al-4V (R56400) alloy [23,24].

Figure 3 presents a comparison of our prepared MEAs with the quinary MEAs prepared in our previous study, indicating that adding Ni not only effectively improved the strength but also maintained the high ductility of the MEAs [23,24]. Additionally, the as-cast Ti65Ni3 MEA exhibited superior mechanical properties when compared with a commercial Ti-6Al-4V alloy. On the other hand, the chemical composition analysis of the Ti65Ni1 and Ti65Ni3 MEAs through EDS is listed in Table 4. For each of the MEAs, five regions were selected for chemical composition analysis and the average values and errors were calculated. The measured compositions were close to the nominal compositions, which ensured the alloy homogeneity and specificity.

The Ti65Ni3 MEA with outstanding mechanical properties was subsequently subjected to TMT to modify its microstructure and achieve features such as fine grains and a heterogeneous structure, which can enhance its mechanical properties. This process is described in the subsequent section.

### 3.2. Performance of the Ti65Ni3 MEA after TMT

**Figure 4:**
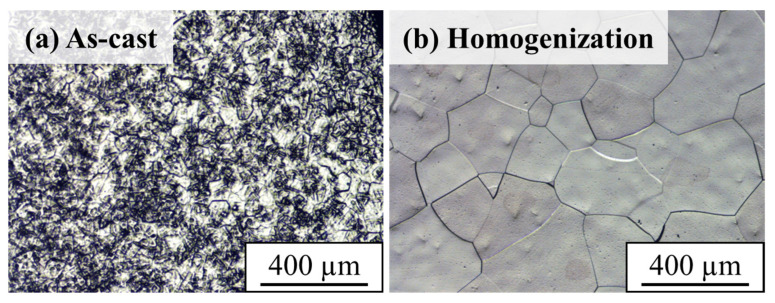
The OM images of (**a**) as-cast (grain size ~66 µm) and (**b**) homogenization (grain size ~133 µm) Ti65Ni3 MEA with different processing.

Before being subjected to TMT, the Ti65Ni3 MEA was homogenized at 1000 °C for 2 h to eliminate composition inhomogeneity and dendrites formed during casting. The grain size of the Ti65Ni3 MEA increased from 66 to 133 µm (Figure 4) after the homogenization process, as determined by means of the line intercept method. Subsequently, the Ti65Ni3 MEA was subjected to two types of rolling processes, CR70 and CR85, followed by annealing for 30, 34, 41, and 63 s at a heating rate of 15 K/s until the temperatures of 743 °C, 770 °C, 817 °C, and 889 °C were reached, respectively.

**Figure 5:**
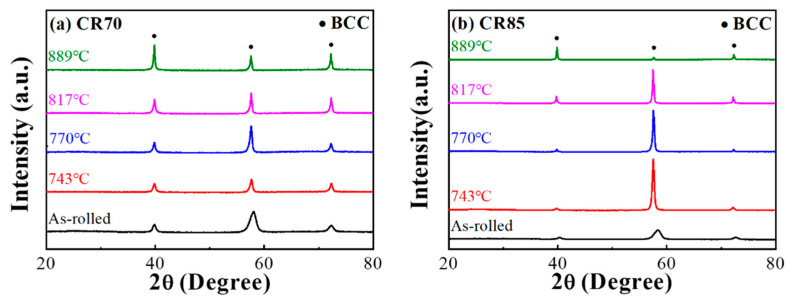
The XRD patterns of (**a**) CR70 and (**b**) CR85 Ti65Ni3 MEA with different annealing temperatures.

**Figure 6:**
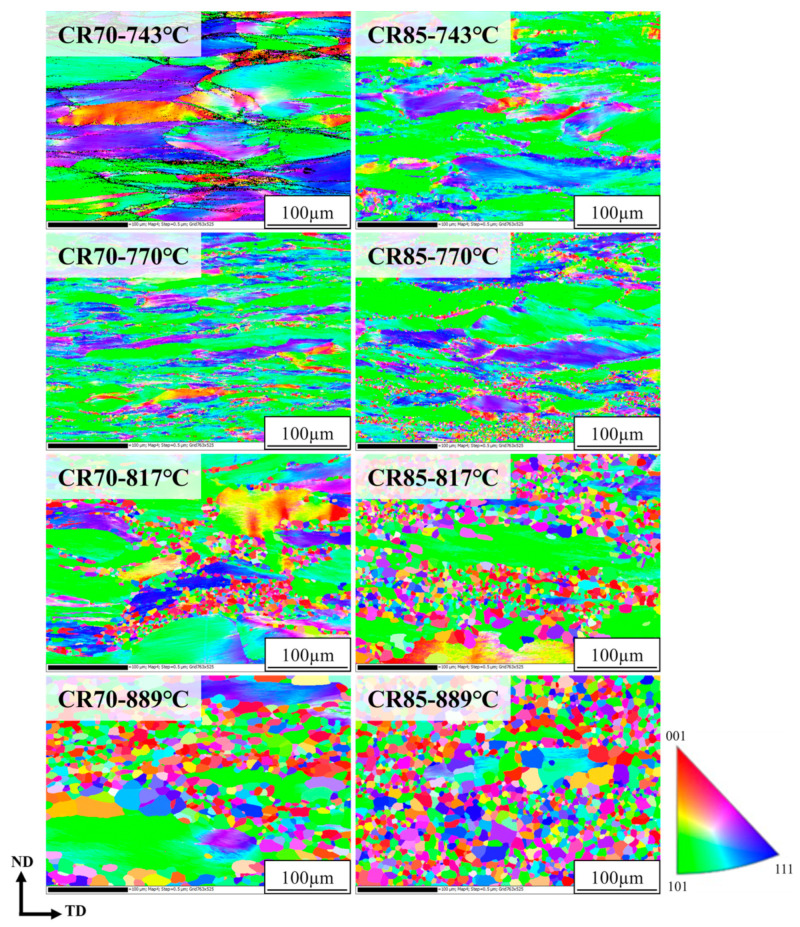
The EBSD images of CR70 and CR85 Ti65Ni3 MEA with different annealing temperatures.

The XRD spectra of the Ti65Ni3 MEA after TMT and rapid annealing contained characteristic peaks that could be attributed to the BCC phase (Figure 5), indicating high solid solution phase stability in both the as-cast and post-TMT states. EBSD analysis was conducted to determine the recrystallization behavior of the Ti65Ni3 MEA after TMT and rapid annealing (Figure 6). The analysis results demonstrated that the recrystallization ratio increased with the annealing temperature. Under the same annealing conditions, the sample exhibited higher recrystallization ratios after the CR85 rolling process than it did after the CR70 process. This can be attributed to the larger plastic strain accumulated through cold rolling, enhancing the recrystallization ability of the MEA. The Ti65Ni3 MEA sample underwent initial recrystallization at 770 °C after CR70 processing and at 743 °C after CR85 processing. Notably, at a given annealing temperature (e.g., 817 °C), the sample exhibited a substantially larger recrystallization area after the CR85 process than it did after the CR70 process. This can be attributed to the larger plastic strain, which resulted in the accumulation of more strain energy, reducing the initial recrystallization temperature. Therefore, compared with the CR70 route, the CR85 route accumulates more strain energy, increasing the number of nucleation sites and reducing the initial recrystallization temperature of the Ti65Ni3 MEA.

**Figure 7:**
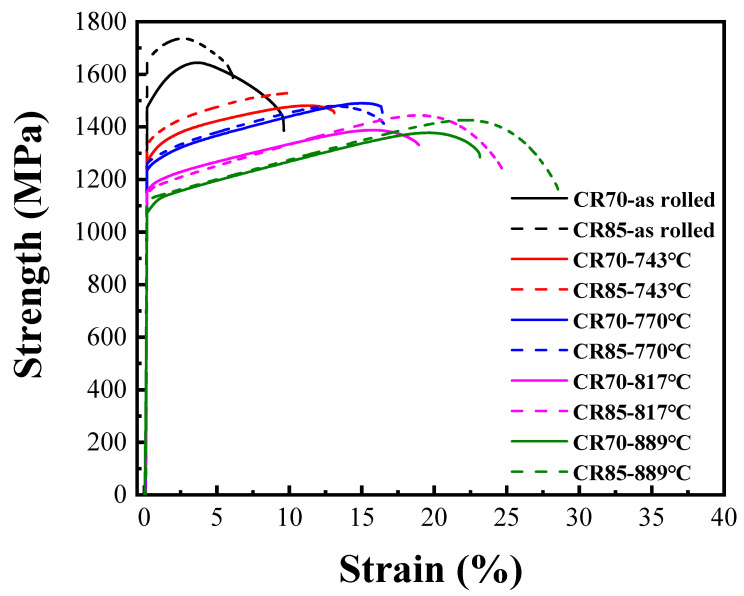
The mechanical tensile stress–strain curves of CR70 and CR85 Ti65Ni3 MEAs with different annealing temperatures.

Figure 7 and Table 5 present the tensile test results obtained for the Ti65Ni3 MEA subjected to TMT. The sample exhibited a yield strength of approximately 1638 MPa and 5.6% plastic strain after the CR85 process, and it exhibited a yield strength of 1478 MPa and 9.5% plastic strain after the CR70 process. Regarding the properties observed after the annealing process executed at a heating rate of 15 K/s, the results revealed that the yield strength of the MEA annealed for 30 s (reaching a sample temperature of 743 °C) substantially decreased to approximately 1270 MPa after the CR70 process and 1351 MPa after the CR85 process. By contrast, the ductility of the sample after the CR70 and CR85 processes increased notably to approximately 13%. Subsequently, the yield strength decreased gradually with increasing annealing time, but the sample’s ductility continued to increase. The yield strength decreased to approximately 1216 MPa after the CR70 process and to 1250 MPa after the CR85 process, and the ductility increased to approximately 16% when the MEA sample was annealed for 34 s (with the sample temperature reaching 770 °C). Subsequently, when the MEA sample was annealed for 41 s (reaching a sample temperature of 817 °C), the yield strength observed after the CR70 and CR85 processes decreased continuously to approximately 1140 MPa. However, the ductility increased to approximately 16% after the CR70 and CR85 processes. The descending slope of yield strength changed slightly because of the presence of recrystallized fine grains, contributing numerous grain boundaries that increased yield strength and compensated for the strain loss due to annealing. Furthermore, when the MEA sample was annealed for 63 s (reaching a sample temperature of 889 °C), its yield strength decreased to approximately 1100 MPa after both the CR70 and CR85 processes, indicating nearly complete recrystallization. By contrast, notably, the ductility of the sample increased to more than 23%.

According to the principles of physical metallurgy, the recrystallization rate of HEAs can be influenced by the following two factors: rapid annealing temperature and strain energy accumulation. A higher annealing temperature can increase the recrystallization rate and accelerate the coarsening of recrystallized grains. Moreover, a larger strain energy accumulation rate can reduce the initial recrystallization temperature and enhance the nucleation rate during recrystallization. In this study, under identical annealing conditions, the Ti65Ni3 MEA sample exhibited higher yield strength and ductility after the CR85 process than it did after the CR70 process. This can be attributed to the increase in plastic strain and strain energy due to heavier cold rolling in the CR85 process, resulting in more nucleation sites and finer grain structures over shorter annealing times. It is considered that because the strain-free grains replace the originally deformed grains, the alloy can withstand more dislocations during deformation that improve the ductility of the MEAs. In addition, according to the Hall–Petch effect, the finer grains can more effectively halt the dislocation movement, thereby enhancing the strength of the alloy.

**Figure 8:**
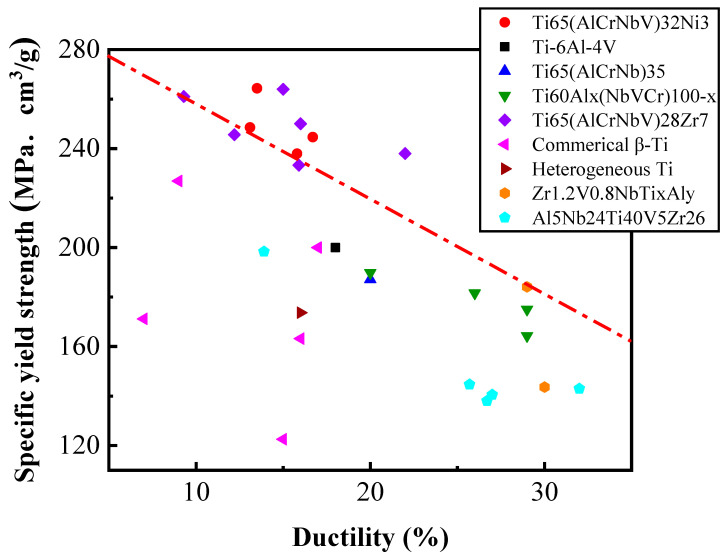
Comparison of specific yield strength and ductility. Dashed line separates the data of the present work and previous studies [15,23,24,29,30,31,32].

In summary, the Ti65Ni3 MEA sample subjected to both the CR70 and CR85 processing routes and annealed for 30 s exhibited higher yield strength (>1250 MPa) and ductility (>13%) than did the other samples prepared in this study. Notably, the Ti65Ni3 MEA subjected to CR85 processing and annealed for 30 s exhibited several outstanding mechanical properties simultaneously, with a yield strength of 1351 MPa, ultimate tensile strength of 1530 MPa, and 13% ductility. After dividing by the density (5.11 g/cm^3^), it presented the high specific yield strength of 264 MPa·cm^3^/g and specific tensile strength of 300 MPa·cm^3^/g. Through thermo-mechanical treatment, the Ti65Ni3 MEA possesses an outstanding synergy of mechanical properties superior to those of commercial Ti alloys and comparable to our previously developed Ti65Zr7 MEA (Figure 8) [15,23,24,29,30,31,32]. In addition, it also demonstrates better specific yield strength than other materials with huge potential in industry application. 

## 4. Conclusions

A series of Ti_x_(AlCrNbV)_100−x−y_Ni_y_ MEAs were successfully synthesized through a process involving arc melting, casting, and TMT. On the basis of our experimental results, we drew the following conclusions regarding the microstructure evolution and mechanical properties of the alloys:

1. The density of the Ti_x_(AlCrNbV)_100−x−y_Ni_y_ series MEAs ranged from 5.02 to 5.12 g/cm^3^, closely aligning with the predetermined target of approximately 5 g/cm^3^. Notably, all MEAs exhibited a single BCC structure in both the as-cast state and after-TMT processing.
2. Microalloying with Ni could not only effectively improve strength but also preserve the favorable ductility in the Ti_65−x_(AlCrNbV)_35_Ni_x_ MEAs. The as-cast Ti65Ni3 MEA exhibited optimal strength and ductility. However, higher Ni content led to the embrittlement of the MEA.
3. The Ti65Ni3 MEA subjected to CR70 and CR85 processing and annealed for 30 s exhibited high yield strength (>1250 MPa) and ductility (>13%). In particular, the Ti65Ni3 MEA subjected to CR85 processing and annealed for 30s exhibited a specific yield strength of 264 MPa·cm^3^/g, specific tensile strength of 300 MPa·cm^3^/g, and ductility of >13%.

## Figures and Tables

**Table 1:** The parameters of the as-cast Ti_x_(AlCrNbV)_100−x−y_Ni_y_ MEAs.

Composition	ΔS (kJ·mol^−1^)	δr (%)	Lattice Constant (Å)
Ti63Ni3	10.26	5.23	3.201
Ti64Ni2	9.99	5.07	3.212
Ti65Ni1	9.68	4.91	3.192
Ti65Ni3	9.92	5.14	3.190
Ti65Ni4	9.99	5.16	3.188
Ti65Ni5	10.30	5.36	3.185

**Table 2:** The density of the as-cast Ti_x_(AlCrNbV)_100−x−y_Ni_y_ MEAs.

Composition	Theoretical Density (g/cm^3^)	Measured Density (g/cm^3^)	Error (%)
Ti63Ni3	5.09	5.06	0.59
Ti64Ni2	5.07	5.10	0.59
Ti65Ni1	5.02	5.04	0.39
Ti65Ni3	5.06	5.11	0.99
Ti65Ni4	5.08	5.10	0.39
Ti65Ni5	5.09	5.10	0.20

**Table 3:** The tensile mechanical properties of the as-cast Ti_65_(AlCrNbV)_35_ and Ti_x_(AlCrNbV)_100−x−y_Ni_y_ MEAs [23].

Composition	Hardness (HV)	Yield Strength (MPa)	Ultimate Strength (MPa)	Ductility (%)
Ti_65_(AlCrNbV)_35_	317 ± 3	921 ± 11	1159 ± 14	25.3 ± 1.4
Ti63Ni3	373 ± 4	1070 ± 29	1167 ± 55	10.7 ± 1.8
Ti64Ni2	364 ± 5	1061 ± 32	1075 ± 58	11.6 ± 2.4
Ti65Ni1	343 ± 7	963 ± 26	1091 ± 72	26.5 ± 2.7
Ti65Ni3	355 ± 6	1065 ± 35	1230 ± 40	17.7 ± 1.5
Ti65Ni4 *	386 ± 12	1145 ± 41	N/A	N/A
Ti65Ni5 ^#^	394 ± 6	N/A	N/A	N/A

Remarks: * The material broke after passing the yielding point. ^#^ The material broke before passing the yielding point.

**Table 4:** Chemical composition of as-cast Ti65Ni1 and Ti65Ni3 MEAs.

Composition		Ti	Al	Cr	Nb	V	Ni
Ti65Ni1	Nominal (at.%)	65	8.5	8.5	8.5	8.5	1
	measured (at.%)	67.49 ± 0.63	6.81 ± 0.44	7.62 ± 0.68	9.03 ± 0.48	8.10 ± 0.41	0.94 ± 0.25
Ti65Ni3	nominal (at.%)	65	8	8	8	8	3
	measured (at.%)	69.11 ± 0.76	6.13 ± 0.42	6.97 ± 0.48	7.30 ± 0.75	7.77 ± 0.19	2.71 ± 0.25

**Table 5:** Tensile mechanical properties of Ti65Ni3 MEA with different TMT parameters.

Processing	CR70			CR85		
Mechanical Properties	Yield Strength	Ultimate Strength	Ductility	Yield Strength	Ultimate Strength	Ductility
	(MPa)	(MPa)	(%)	(MPa)	(MPa)	(%)
As-rolled	1478 ± 14	1649 ± 21	9.5 ± 0.2	1638 ± 12	1724 ± 11	5.6 ± 0.7
743 ℃	1270 ± 9	1485 ± 18	13.1 ± 1.6	1351 ± 17	1530 ± 22	13.5 ± 2.3
770 ℃	1216 ± 13	1421 ± 12	15.8 ± 2.4	1250 ± 14	1478 ± 13	16.7 ± 3.3
817 ℃	1146 ± 6	1390 ± 14	18.8 ± 1.2	1139 ± 17	1480 ± 14	21.5 ± 1.7
889 ℃	1070 ± 2	1374 ± 9	23.2 ± 2.7	1115 ± 14	1427 ± 21	27.1 ± 1.0

## Data Availability

Data are contained within the article.
